# PLA-lignin nanofibers as antioxidant biomaterials for cartilage regeneration and osteoarthritis treatment

**DOI:** 10.1186/s12951-022-01534-2

**Published:** 2022-07-16

**Authors:** Ruiming Liang, Xingchen Yang, Pek Yin Michelle Yew, Sigit Sugiarto, Qiang Zhu, Jinmin Zhao, Xian Jun Loh, Li Zheng, Dan Kai

**Affiliations:** 1 Guangxi Engineering Center in Biomedical Materials for Tissue and Organ Regeneration, International Joint Laboratory on Regeneration of Bone and Soft Tissues, Guangxi Key Laboratory of Regenerative Medicine & Collaborative Innovation Center of Regenerative Medicine and Medical Biological Resources Development and Application , Life Sciences Institute, Guangxi Medical University, Nanning, 530021 China; 2 Institute of Materials Research and Engineering (IMRE), A*STAR, 2 Fusionopolis Way, #08-03 Innovis, Singapore, 138634 Singapore; 3 Department of Biomedical Engineering, Faculty of Engineering, National University of Singapore, Singapore, 117583 Singapore; 4 Department of Orthopaedics Trauma and Hand Surgery, Guangxi Key Laboratory of Regenerative Medicine, The First Affiliated Hospital of Guangxi Medical University, Guangxi Medical University, Nanning, 530021 China; 5 Institute of Sustainability for Chemicals, Energy and Environment (ISCE2), A*STAR, 2 Fusionopolis Way, Innovis, #08-03, Singapore, 138634 Singapore

**Keywords:** Tissue engineering, Free radicals, Antioxidant, Stem cell differentiation

## Abstract

**Background:**

Osteoarthritis (OA) is common musculoskeletal disorders associated with overgeneration of free radicals, and it causes joint pain, inflammation, and cartilage degradation. Lignin as a natural antioxidant biopolymer has shown its great potential for biomedical applications. In this work, we developed a series of lignin-based nanofibers as antioxidative scaffolds for cartilage tissue engineering.

**Results:**

The nanofibers were engineered by grafting poly(lactic acid) (PLA) into lignin via ring-opening polymerization and followed by electrospinning. Varying the lignin content in the system was able to adjust the physiochemical properties of the resulting nanofibers, including fiber diameters, mechanical and viscoelastic properties, and antioxidant activity. In vitro study demonstrated that the PLA-lignin nanofibers could protect bone marrow-derived mesenchymal stem/stromal cells (BMSCs) from oxidative stress and promote the chondrogenic differentiation. Moreover, the animal study showed that the lignin nanofibers could promote cartilage regeneration and repair cartilage defects within 6 weeks of implantation.

**Conclusion:**

Our study indicated that lignin-based nanofibers could serve as an antioxidant tissue engineering scaffold and facilitate the cartilage regrowth for OA treatment.

**Graphical Abstract:**

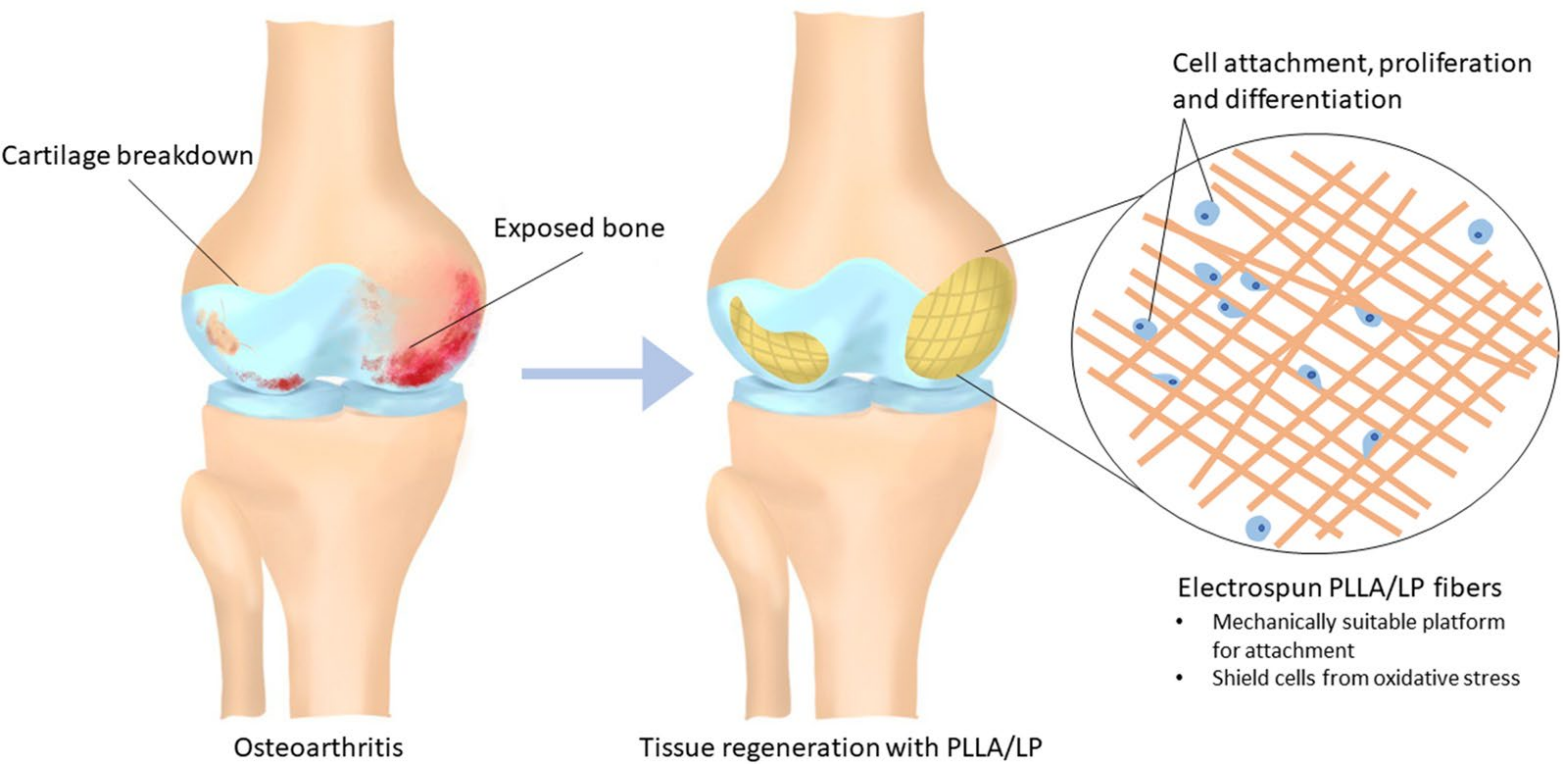

**Supplementary Information:**

The online version contains supplementary material available at 10.1186/s12951-022-01534-2.

## Background

Osteoarthritis (OA) is a typical musculoskeletal disease caused by progressive degeneration of cartilage, [1] Articular cartilage may undergo the loss of cellularity and degradation of the cartilage extracellular matrix (ECM), caused by inflammatory diseases, trauma, or aging. Despite recent advances in cartilage tissue engineering (TE) and stem cell therapy, the treatment for OA remains a challenge today. Native cartilage lacks the ability of regeneration: chondrocytes show poor proliferative capability and cartilage is an avascular tissue, which hinders the immigration of stem cells, [2]. Moreover, OA always induces oxidative stress and generates free radicals, such as reactive oxygen species (ROS). [3] The local accumulation of ROS limits the self-repair ability of cartilage tissue by increasing its catabolism while decreasing the anabolism [4]. Excess ROS also stimulates the apoptosis of native chondrocytes and accelerates the degradation of cartilage ECM, by increasing the lipid peroxidation, damaging the ion transporters on cell membrane and reducing synthesis of proteoglycans [5]. It is important to note that the oxidative environment can also interfere with the chondrogenic differentiation of stem cells. Kisiday et al. found that lower level of ROS promoted mesenchymal stem cells (MSCs) chondrogenesis and increased collagen accumulation [6].

Antioxidants are considered as effective agents for the treatment of OA. For example, sulforaphane is able to protect chondrocytes against cell death induced by oxidative stress environment [7]. While vitamin E, a chain-breaking antioxidant, demonstrates strong chondroprotective effects and improved the therapeutic efficacy of MSCs against hydrogen peroxide-induced oxidative stress for OA treatment [8, 9].

Besides using small antioxidant molecules, development of antioxidant TE scaffolds can be a state-of-art approach to improve cartilage regeneration against oxidative stress. Herein, we propose lignin-based nanofibrous scaffolds with antioxidant properties for cartilage TE. Lignin is the most abundant phenolic biopolymer in nature. Its unique chemical structure together with large numbers of guaiacyl and syringyl phenolic units endow lignin with strong antioxidant activities [10]. Lignin has been widely studied as an antioxidant for plastics, rubber, elastomers and composites. However, lignin as an antioxidant for biomedical applications is rarely studied. In fact, lignin exhibits several attractive features as a biomaterial, including antioxidant activity, biodegradability, and capability of absorbing ultraviolet (UV) radiation [11–14]. Various lignin copolymers, such as lignin-poly(ethylene glycol), lignin-polycaprolactone, lignin-polyhydroxybutyrate, were synthesized via atom transfer radical polymerization (ATRP) or ring-opening polymerization [15–19]. These polymers have demonstrated excellent mechanical properties, decent antioxidant activity and biocompatibility in vitro, potentially for biomedical applications. However, their bioactivity in vivo is still unclear.

In this study, we grafted poly (lactic acid) (PLA) onto lignin and synthesized a series of lignin-PLA(LP) copolymers via ring-opening polymerization. PLA is a biodegradable and biocompatible aliphatic polyester, and it have been widely used as a promising biomaterial for multiple biomedical applications. In our previous study, we have demonstrated that grafting PLA onto lignin-PLA could improve the miscibility of lignin in the nanofibers for mechanical reinforcement, and the composite nanofibers also displayed good biocompatibility in vitro with multiple cell lines [19]. Here, to further explore its potential application in tissue regeneration, we fabricated nanofibrous TE scaffolds based on the lignin copolymers via electrospinning. The mechanical and viscoelastic properties, as well as antioxidant activity of the scaffolds were characterized. Moreover, the chondrogenesis of MSCs on nanofibrous scaffolds and cartilage repair were carried out to evaluate its potential for cartilage TE against oxidative stress for OA treatment.

## Results and discussion

### Fabrication and characterization of PLLA/LP nanofibers

**Fig. 1:**
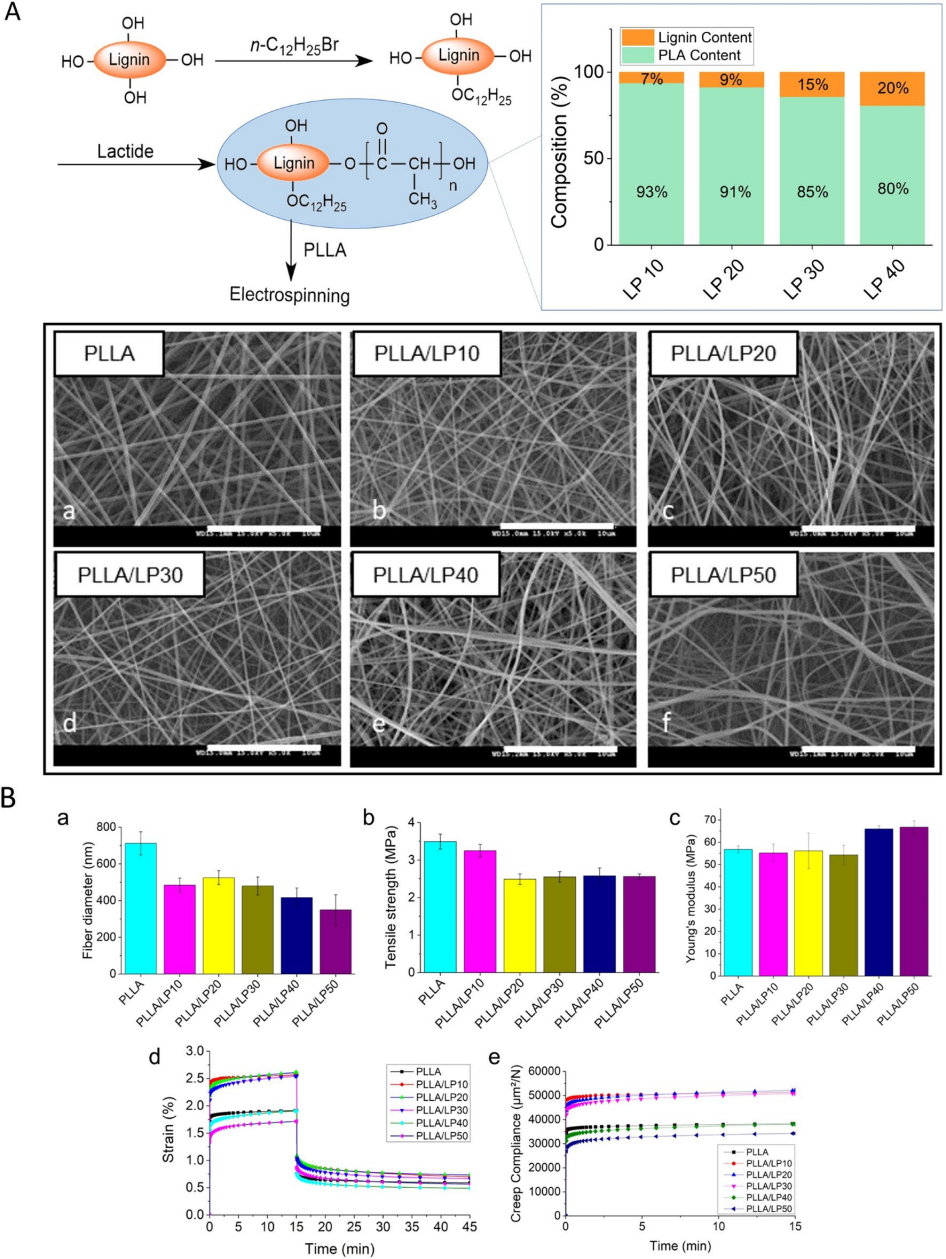
**A** Synthesis steps of lignin-PLA (LP) copolymers with the corresponding copolymer compositions, and the morphology of electrospun PLLA/LP nanofibers. **B** Characterization of PLLA/LP nanofibers: **a** Fiber diameter; **b** Tensile strength; **c** Young’s modulus; **d** creep and recovery strain–time curves and **e** Creep compliance–time curves

In this study, lignin was grafted with PLA (Fig. 1A) to improve its miscibility in Poly-L-lactic acid (PLLA) matrix. Various lengths of PLA were grafted onto lignin by varying the feeding ratio, and the molecular weight of the copolymers ranges from 75.6 kDa for LP10 to 28.5 kDa for LP50 (Additional file 1: Table S1, Figure S1, S2). The resulting copolymers exhibits different fractions of PLA and lignin (calculated based on the results from NMR and GPC). At higher feed ratio of lignin to PLA, the resulting lignin content in the copolymer increases. The LP10 has the lowest lignin content of 7%, while LP40 exhibit the highest at 20% (Fig. 1A). It is worthy to note that the dodecylation pretreatment of lignin was employed to facilitate the ring-opening polymerization and improve matrix/additive compatibility. But the process is relative energy-intensive and may cause environmental concern (due to the halogenated compound). In future work, sustainable and green methods need to be developed to synthesize novel lignin copolymers while protecting the phenolic hydroxyl groups to retain the antioxidant activities.

Such LP copolymers were blended with PLLA and electrospun into nanofibrous scaffolds for cartilage TE. As shown in Fig. 1A, all the fibers exhibited uniform and bead-free nanoscale morphology, and the fiber diameter ranges from 712 ± 63 nm for neat PLLA to 350 ± 80 nm for PLLA/LP50. The results showed that the addition of LP copolymers reduce the fiber diameter (Fig. 1B(a)), probably due to the lower molecular weight of such copolymers, which causes a decrease of viscosity of the spinning solution [20, 21]. The mechanical properties of the nanofibrous scaffolds were carried out by tensile test. The tensile strength and Young’s modulus of the fibers were summarized in Fig. 1B (b and c). The addition of LP copolymers decreased the tensile strength of the nanofibers from 3.49 ± 0.20 MPa for PLLA to 2.49 ± 0.14 MPa for PLLA/LP20, but it increased the Young’s Modulus from 56.8 ± 1.6 for PLLA to 66.8 ± 2.8 MPa for PLLA/LP50 (Additional file 1: Figure S5). In Fig. 1B(b), the nanofibers exhibit comparable tensile strength with the native articular cartilage (0.8- 25 MPa), while the Young’s modulus (Fig. 1B(c)) falls within the range of 5–25 MPa of the native articular cartilage [22]. The results indicate that our nanofibers could be a suitable scaffold with comparable mechanical properties for cartilage repair.

The viscoelastic behaviour of the nanofibrous scaffolds was evaluated by creep-recovery experiment. Creep-recovery test is the most effective method to analyse the elasticity of polymeric scaffold. In a typical creep test, a constant stress is applied onto the sample and time-dependent deformation (strain) is detected. As shown in Fig. 1B(d), all scaffolds display a typical viscoelastic creep-recovery curve following Kelvin-Voigt model. In the model, the material is represented by a Hookean spring and a Newtonian dashpot in parallel. Compared to neat PLLA fibers, PLLA/LP10, PLLA/LP20 and PLLA/LP30 showed higher creep compliance levels. On the other hand, LP40 and LP50 slightly decreased creep compliance of the resulting nanofibers.

The interaction between PLLA matrix and LP fillers play a crucial role to influence the viscoelastic properties of the composite nanofibers. Here, there are two types of interactions between PLLA and LP copolymers: 1) polymer chain inter-entanglement between PLLA matrix and PLA polymer chains on the surface of LP; 2) hydrogen-bonding interactions between C = O of PLLA and OH groups on lignin. In such case, the polymer chain inter-entanglement is dominate in the nanofiber system, and therefore, those LP copolymers with longer PLA chain length were more compatible with PLLA matrix, which enable the softening of PLLA and facilitate the polymer chain movement in the amorphous regions of the matrix.

### Antioxidant activity of PLLA/LP nanofibers

**Fig. 2:**
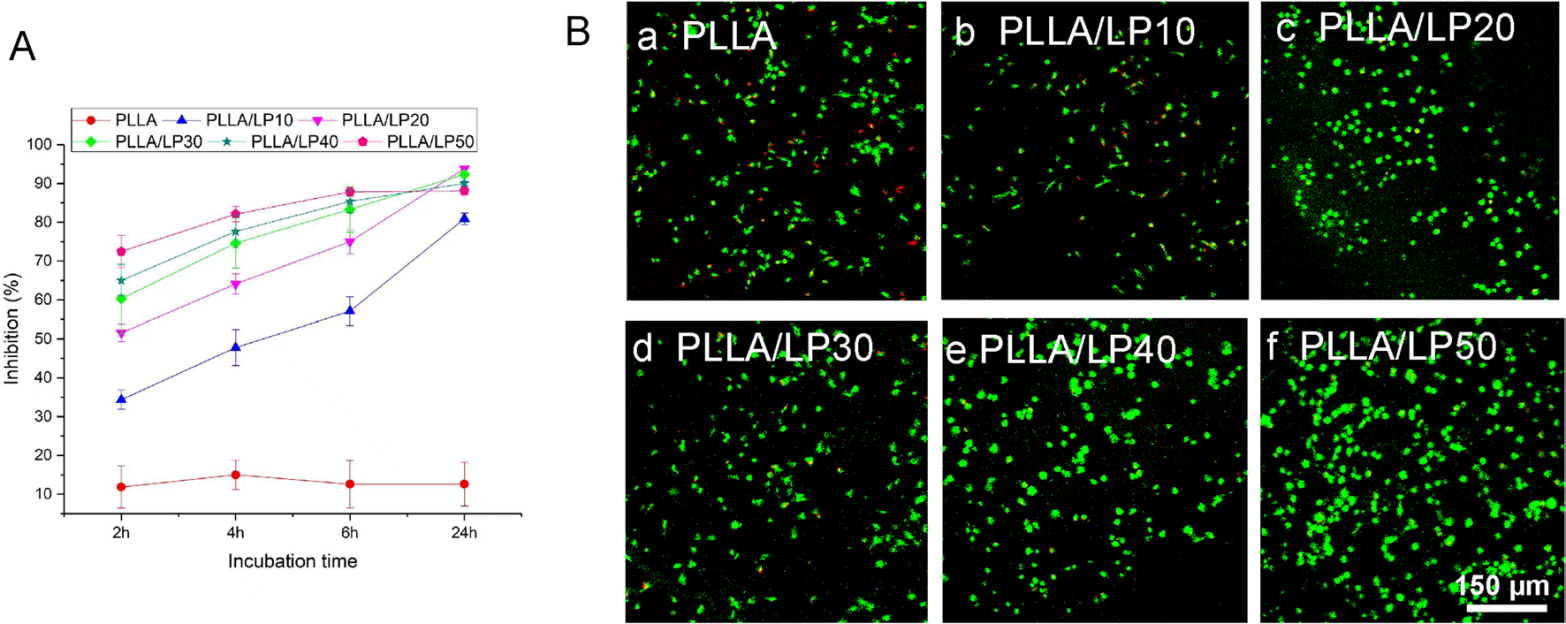
**A** Free radical inhibition (antioxidant activity) of PLLA and PLLA/LP nanofibers by DPPH assay. **B** The viability of BMSCs on the antioxidant nanofibers of PLLA/LP with H_2_O_2_ exposure

Oxidative stress is a major impediment against cartilage repair, and antioxidants could play a crucial role in addressing this issue. Lignin is a natural antioxidant from plants. The antioxidant activities of PLLA/LP nanofibers were evaluated by 1,1-diphenyl-2-picrylhydrazyl (DPPH) assay. Neat PLLA fibers were used as negative control, respectively. As shown in Fig. 2A, PLLA fibers showed very low antioxidant activity (< 15% free radical inhibition) during the first 24 h. The addition of lignin copolymers into PLLA improved the antioxidant activity of the resulting nanofibers, and the nanofibers with higher lignin content exhibited higher free radical inhibition. It is worthy to note that the free radical inhibition of PLLA/LP nanofibers increased gradually with time, and after 24 h of incubation, the free radical inhibition values of these composite nanofibers (except PLLA/LP10) reached more than 90%. It is report that the half-life of small antioxidant molecules (such as vitamins) in the human body is relatively short (normally < 30 min) [23], indicating that such antioxidants loses its function very fast. Comparatively, our lignin copolymers showed a slower response against DPPH radicals but reached the same level of antioxidant activity at 24 h, indicating such LP copolymers have longer half-life and more stable in the oxidative stress environment.

The antioxidative properties of PLLA/LP nanofibers are further evaluated through the viability of bone marrow mesenchymal stem cells (BMSCs)in the exposure of H_2_O_2_ (24 h) and was confirmed with the live/dead assay. As revealed by the confocal imaging (Fig. 2B), H_2_O_2_ treatment resulted in the highest number of dead cells (red) and the lowest number of live cells (green) observed in Figure B(a) of PLLA. The PLLA/LP studies exhibited higher live to dead cell ratio in the H_2_O_2_ environment. The PLLA/LP10 exhibited a small number of dead cells but moving forward across PLLA/LP20-50 B (b-f), there are no signs of the red markers, a significant difference to PLLA. These observations indicates that PLLA/LP nanofibers are able to shield BMSCs from oxidative stress. The presence of LP had a significant impact on the antioxidative properties in the fibers. Lignin has been consistently studied and exploited for its antioxidative properties. The various functional groups exist in this complex heteropolymer such as the methoxy, phenolic hydroxyl, and double bond groups, plays a crucial role as a free radical scavenger [13, 24].

### Morphology and proliferation of BMSCs on PLLA/LP nanofibrous scaffold

**Fig. 3:**
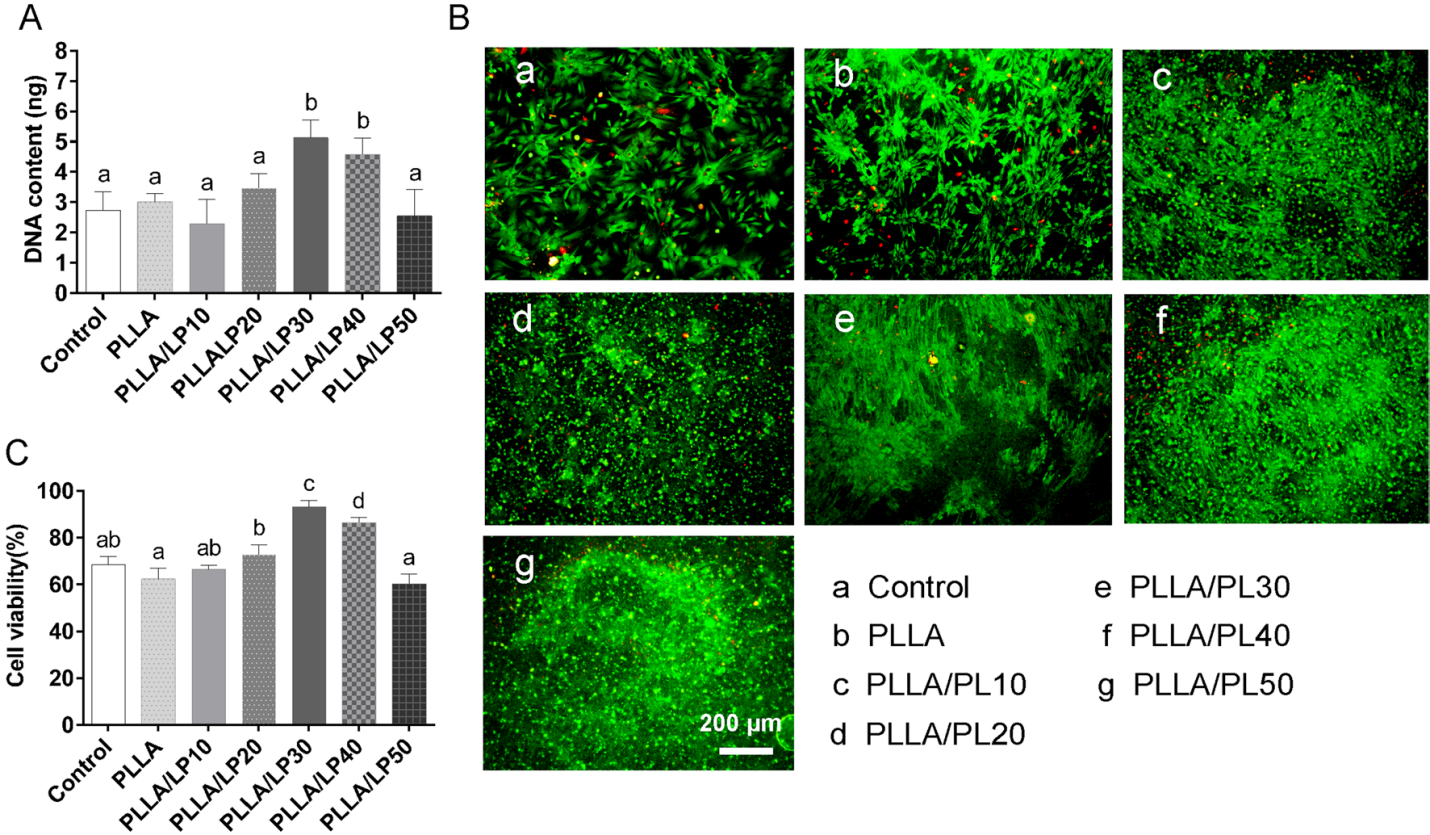
**A** The proliferation of BMSCs on PLLA/LP the nanofibers. **B** Confocal imaging of live (green) and dead (red) cells. (C) Quantification of cell viability. Values are presented as the means ± SD, n = 3, different letters denote significances with P < 0.05

The proliferation of BMSCs on PLLA/LP the nanofibers was evaluated by its resulting deoxyribonucleic acid (DNA) contents. In Fig. 3A, PLLA/LP30 exhibited the highest DNA content as compared to the rest of the PLLA/LP. A calcein/ propidium iodide (PI) live/dead viability assay kit determines the cell viability based on plasma membrane integrity and esterase activity of BMSCs. As shown in Fig. 3B-C, the PLLA/LP nanofibers supported a higher survivability of BMSCs than PLLA nanofibers at day 21. In general, PLLA exhibits good biodegradation and biocompatibility but poor bioactivity due to the lack of functional groups [25]. The strategic optimization of electrospinning condition and PLLA/LP blend would produce nanofibers that exhibit suitable functionality and scaffold structure to facilitate tissue regeneration. The significant increased cell proliferation and viability on PLLA/LP30 demonstrates the optimal nanofibers characteristics to facilitate tissue regeneration. Saudi et al. had also reported electospun poly (glycerol sebacate)-poly(vinyl alcohol) fibers with lignin had promoted the neural cell proliferation and differentiation [26]. The addition of lignin in the blend induces anti-oxidative properties that would facilitate cell viability [27, 28]. The low cell viability at higher lignin concentrations is possibly due to the inhibition from lignin itself pass the ideal range. At excessive amounts of lignin, the cytotoxicity characteristics becomes a detrimental factor, as observed between PLLA/LP50 and the control. Ugartondo et al. reported cytotoxicity effects of lignin to human keratinocytes and murine fibroblast 3T3 cells but at high concentrations [29].

**Fig. 4:**
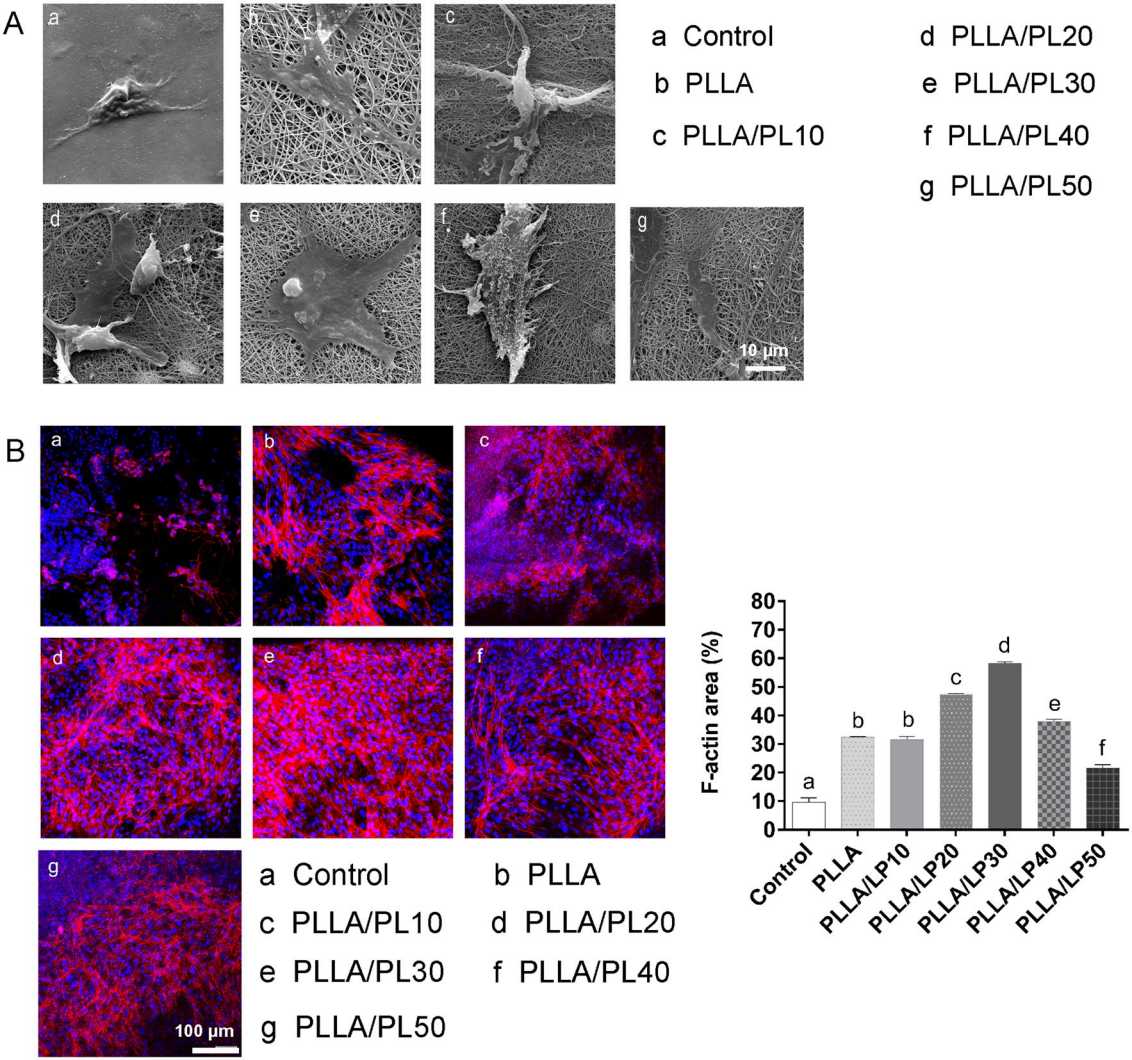
**A** The morphology of BMSCs on PLLA/LP nanofibers were observed by SEM. **B** Cytoskeletal morphology of BMSCs were investigated using rhodamine-phalloidin staining for actin cytoskeleton. Values are presented as the means ± SD, n = 3, different letters denote significances with P < 0.05

The morphology of BMSCs on PLLA/LP nanofibers was observed using scanning electron microscopy (SEM) (Fig. 4A). BMSCs have been widely studied with for bone tissue engineering because of its ability to differentiate into chondrocytes, and osteoblasts [30]. The optimal micro-environment of a scaffold is key to facilitate stem cell attachment and proliferation. In the control group, cells exhibited a stretched, long, spindle-shaped, fibroblast-like morphology during the culture period. On day 7, a portion of the cells on PLLA nanofibers exhibit flat and spindle-like characteristics. However, the BMSCs on the PLLA/LP nanofibers were spread out smoothly, and the cells on the PLLA/LP30 gradually became more spherical, which displayed chondrocyte-like morphology. The results indicate that the topography of PLLA/LP nanofibers, especially PLLA/LP30, would be suitable to support cell adhesion [16].

The cytoskeletal morphology was investigated using rhodamine-phalloidin staining for actin filament (F-actin) (red) and 4',6-diamidino-2-phenylindole (DAPI) staining for nuclei (blue) followed by confocal microscopy (Fig. 4B). The actin filamentous network structure is significant to identify the progression of cytoskeleton network. Through the staining, a small number of polymerized actin filaments was observed in the BMSCs of the control group, followed by increasing fluorescent activation in PLLA and PLLA/LP. The presence of F-actin (red) and nuclei is significantly higher in the PLLA/LP group (highest with PLLA/LP30) compared to PLLA, thus indicating better cell proliferation and viability towards chondrogenesis. A similar observation had been reported previously whereby, lignin-PCL nanofibers exhibit higher cell viability of human chondrocytes at 64% compared to just 17% of PCL nanofibers alone [31]. The cytoskeletons of the cells exhibit a wide branched network of elongated actin that would facilitate to achieve better cell extension and growth in the environment. A recent study of lignin/PCL-hydroxyapatite composite had reported an observation of elongated filopodia formed on the film that indicating the maturity of the cell growth stage towards their innate environment [32].

### Chondrogenic differentiation of BMSCs on PLLA/LP nanofibers

**Fig. 5:**
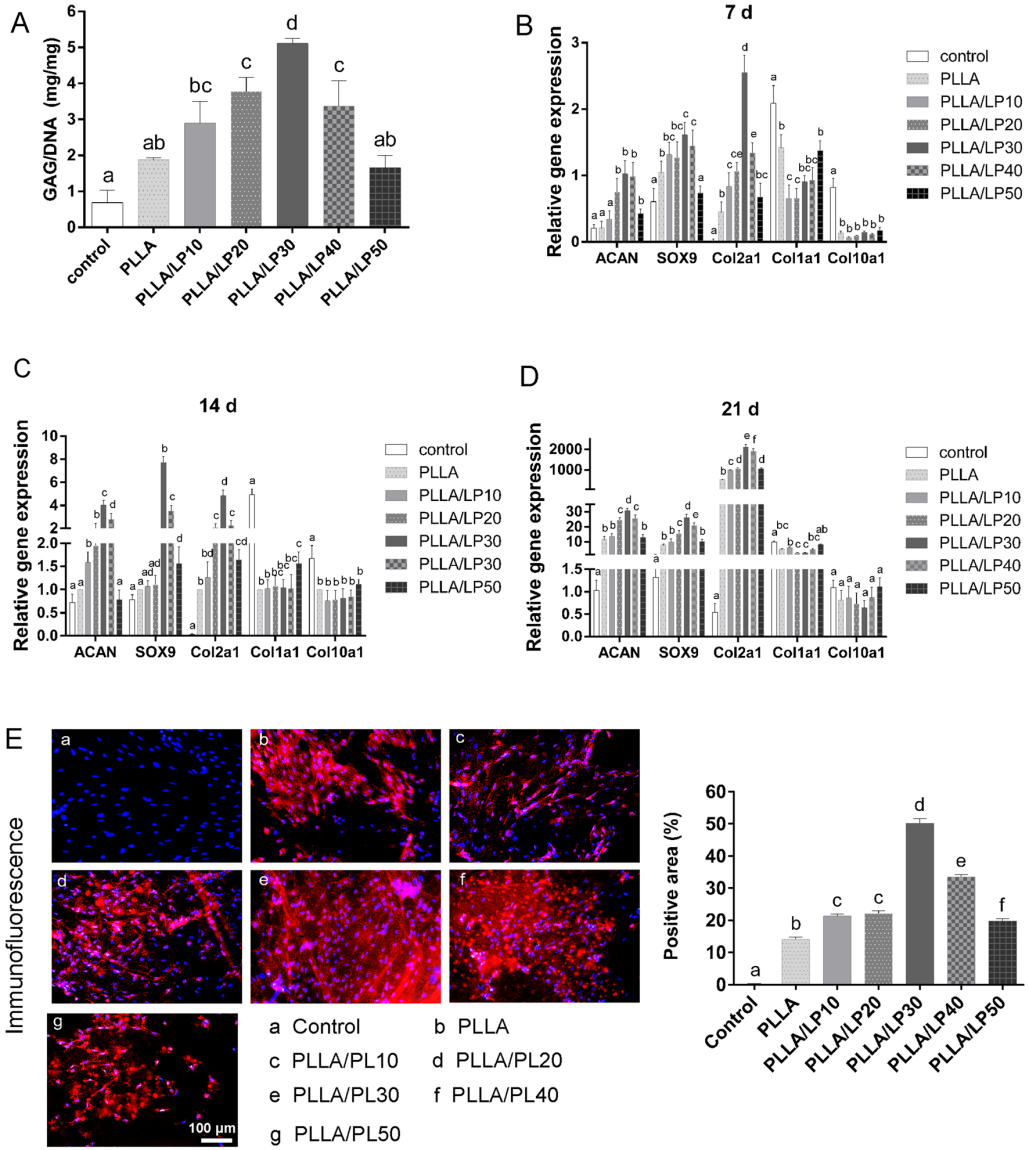
**A** Quantification of GAG secretion of BMSCs by DMMB assay. **B**–**D** qRT-PCR was performed to detect the chondrogenic-related genes expression of *Col2a1, ACAN, SOX9, Col1a1*and *Col10a1*. **E** The secretion of collagen type II (Col2a1) was detected by immunofluorescence. Values are presented as the means ± SD, n = 3, different letters denote significances with P < 0.05

The glycosaminoglycans (GAG) secretion of BMSCs cultured on PLLA/LP nanofibers was evaluated by using 1,9-dimethylmethylene blue (DMMB) assay. GAG is a key marker to identify the process of chondrogenesis and cartilage formation [33]. As shown in Fig. 5A, the GAG production in PLLA/LP groups were higher than that in PLLA group at 21 days, which was increased by 54.29% for PLLA/LP10, 101.07% for PLLA/LP20, 172.56% for PLLA/LP30, and 79.49% for PLLA/LP40. These results indicated that PLLA/LP scaffold had positive effects on GAG secretion for BMSCs. This is important to promote the favorable differentiation of BMSCs at the early stage that will be coupled with the expression of chondrogenic-specific genes [34].

The chondrogenic differentiation of the cells were evaluated through quantitative real-time polymerase chain reaction (qRT-PCR) detection of chondrogenic-related genes expression in BMSCs for 21 days. The *collagen type II* (*Col2a1*), *aggrecan (ACAN),* and *SRY (sex determining region Y)-box9 (Sox9), collagen type I (Col1a1) and collagen type X (Col10a1)* are key genes to distinguish chondrogenesis [35–37]. As shown in Fig. 5B–D, the PLLA/LP nanofibers upregulated the gene expression levels of BMSCs compared with PLLA membrane, in especially PLLA/LP30 group. The expression of gene levels on PLLA/LP30 group in 21 days were all higher than that of PLLA group: nearly 300.73% higher for *Col2a1*, 164.49% higher for *ACAN*, and 234.09% higher for *SOX9*. The slow and minimal increase of *Col10al* throughout the days are the result of its typical expression at a later stage of chondrogenesis towards cartilage maturation and hypertrophy [33]. The low expression of *Col10al* in PLLA/LP30 indicates that the cell growth is further away from chondrocyte hypertrophy [38]. This is also observed in *Col1a1*, where it’s downregulated by the 21 days, with PLLA/LP20 and 30 exhibiting the lowest expression. These results suggest that PLLA/LP synergistically enhances chondrogenic differentiation of the BMSCs.

The secretion of collagen type II (Col2a1), which is specific for cartilage, was detected by immunofluorescence. As shown in Fig. 5E, PLLA/LP nanofibers also showed a significantly increased accumulation of Col2a1 after 21 days of culture than PLLA. Moreover, among all the groups, BMSCs on PLLA/LP30 produced the most abundant extracellular matrix that was rich in Col2a1. The abundance of Col2al indicates primary growth in chondrocytes which is consistent within the cartilaginous tissues [16, 37].

### *PLLA/LP nanofibers promoted cartilage regeneration *in vivo

**Fig. 6:**
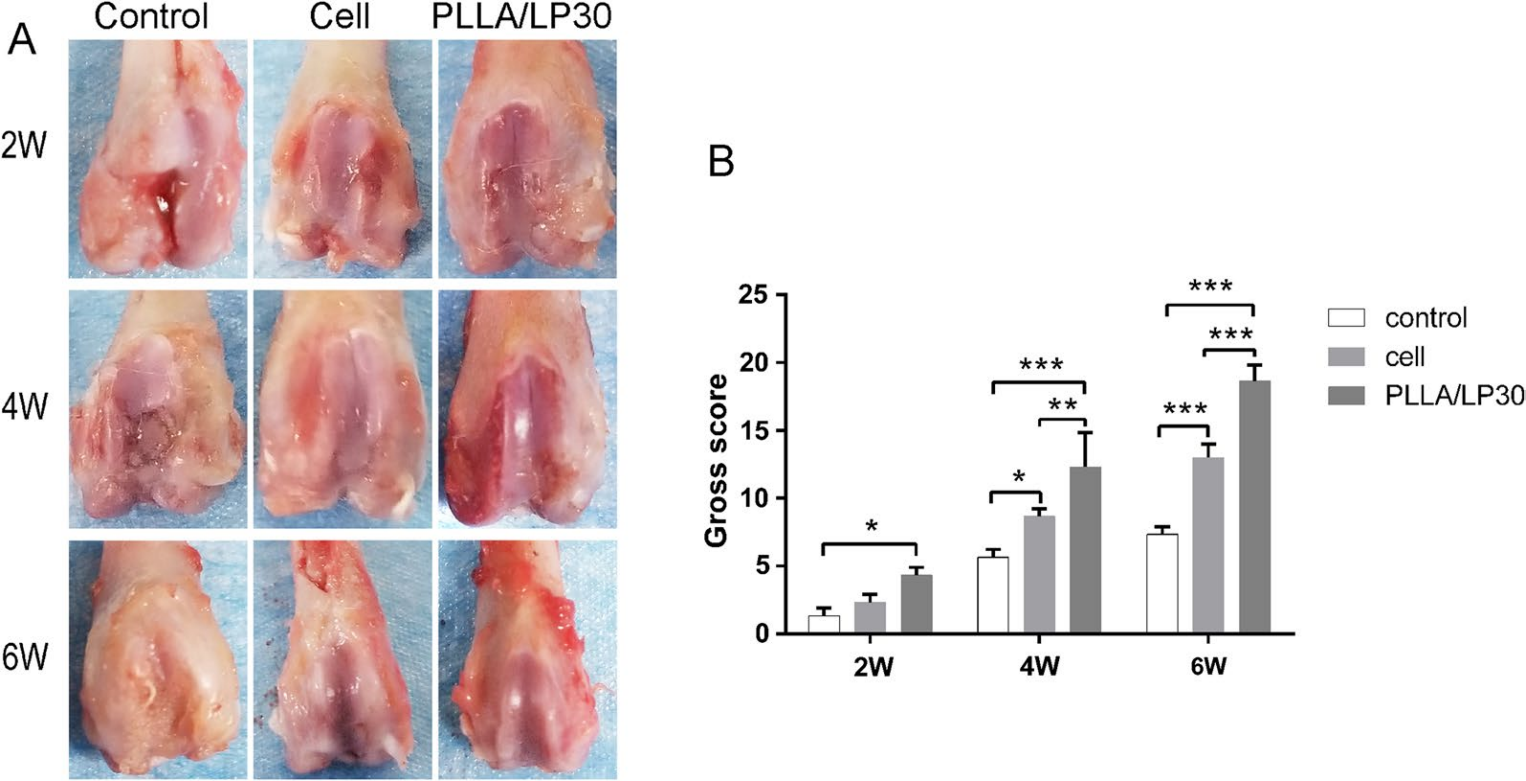
**A** Macroscopic observations and **B** macroscopic score after treatment of nanofibrous membrane on the joint. * denotes *p* < 0.05, ** denotes *p* < 0.01, *** denotes *p* < 0.001 comparison between groups

To assess the ability of cartilage regeneration in vivo, the PLLA/LP30 nanofibers were introduced on the surface of the patellar groove of Sprague-Dawley(SD) rat femurs. No synovial hyperplasia and inflammation in each group was observed after 6 weeks treatment on the knee joints (Fig. 6A). After 6 weeks of post-transplantation, only a few reparative tissues in the defect of the control group, and there were distinct boundary between regenerated neo-tissue and original cartilage. The defects in the control group with BMSCs only contained a few regenerated neo-tissue that are related to the surrounding cartilage. In contrast, the repaired neo-tissue tissues observed in the PLLA/LP30 group exhibited compacted and well-integrated with the neighboring cartilage tissue. We further identified the macroscopic observations by the International Cartilage Repair Society (ICRS) scores. The PLLA/LP30 group showed the highest ICRS scores among all the groups, 12.33 ± 2.05 and 18.67 ± 0.94 on week 4 and 6 week 6, respectively (Fig. 6B).

**Fig. 7:**
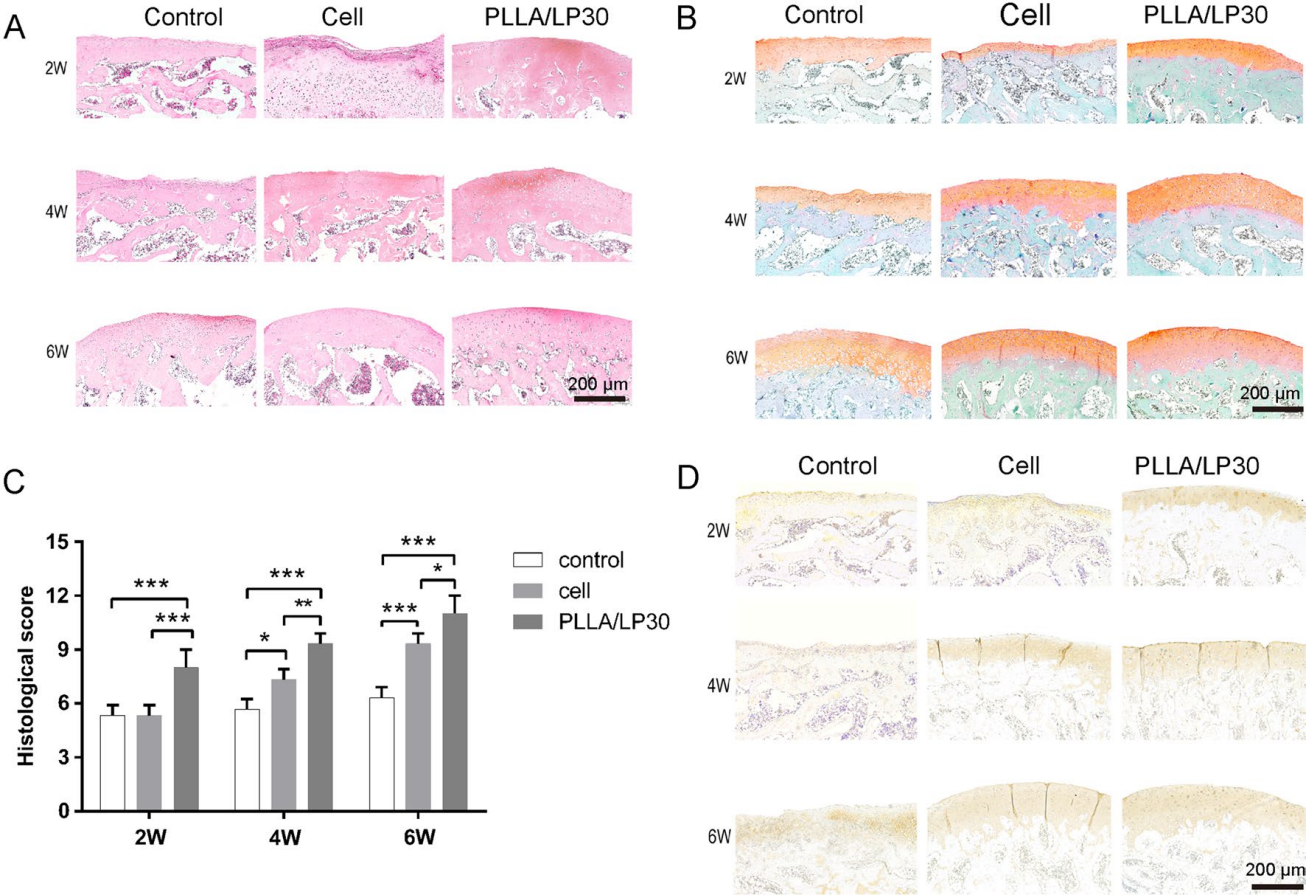
**A** HE staining. **B** safranin O-fast green staining. **C** histological scores. **D** Immunohistochemical staining of collagen type II. * denotes *p* < 0.05, ** denotes *p* < 0.01, *** denotes *p* < 0.001 comparison between groups

Histological evaluation with hematoxylin and eosin (HE) and safranin O-fast green staining were performed after the surgery treatment (Fig. 7A, B). After 6 weeks, the control group exhibited reparative neo-tissues, primarily fibrous tissue with a loose and detached interface to the surrounding cartilage. On another hand, the PLLA/LP30 with cells exhibited the formation of well-integrated hyaline cartilage surrounding the affected cartilage area. The results are here aligned with the GAG and gene expression exhibited with the DMMB assay and qRT-PCR. Specifically, the regenerated cartilage in the PLLA/LP30 group exhibited a more uniform and compact tissue than the other groups, which exhibit minimal differences from the existing surrounding cartilage tissue.

These results were additionally confirmed by the quantitative histological scores (Fig. 7C). At post-transplantation week 4 and 6, the mean score of the defect treated in the PLLA/LP30 group was higher than that treated using only BMSCs. The histological score in the PLLA/LP30 group were 9.33 ± 0.47 and 11.00 ± 0.82 on week 4 and 6, respectively. Immunohistochemistry staining was performed to confirm the levels of collagen type II in the repaired tissue (Fig. 7D). The results presented an almost negative staining in the untreated groups. After 6 weeks of treatment, only a few positive staining was found in the control with BMSCs. A stronger positive staining indicated favorable tissue regeneration in the PLLA/LP30 group after 4- and 6-weeks post-transplantation as compared to other groups.

## Conclusion

In this study, we developed a PLLA/LP nanofibrous scaffold as a suitable platform to facilitate cell growth and development of a native environment to mimic cartilage tissue matrix for tissue regeneration. The composite enables the attachment and cell proliferation of BMSCs due to optimally designed scaffold. PLLA/LP30 nanofibers exhibits good anti-oxidation properties and favorable gene expressions of *SOX9*, *ACAN*, and *Col2al*, while balancing with late stage chondrogenesis gene expression of *Col10al*. Moreover, the successful chondrogenesis and distribution of cells have been proven with the cell studies as well as the in a SD rat cartilage defect model. These favorable characteristics are the hallmark for treating OA through cartilage regeneration. The current study has provided a foundation in fabricating a 3D matrix scaffold suitable for reconstruction. The incorporation of lignin demonstrates its promising capability in terms of its superior anti-oxidative properties and good biocompatibility. This can be applied not only in the biomedical fields but in many others such as consumer care and UV filtration. Lignin is a versatile polymer that can be extremely beneficial when appropriately exploited in the application.

## Materials and methods

### Materials

Alkali lignin was purchased from Tokyo Chemical Industry, and lignin was dried at 105 °C overnight to remove moisture before using. All other chemicals were purchased from Sigma-Aldrich and used as received except where noted.

### Synthesis of lignin-PLA copolymers

The synthesis of PLA-grafted lignin copolymers followed the methods reported in our previous study [39]. Briefly, the copolymers were obtained in a two-step reaction as shown in Fig. 1. First, alkali lignin (5 g) were reacted with *n*-C_12_H_25_Br (7.5 g) in aqueous solution in the presence of K_2_CO_3_ (4 g) and *n*-Bu_4_NBr (5 mg). After refluxing with stirring for 2.5 days at 130 °C, the mixture was cooled down and the dodecylated lignin precipitate was obtained after filtration and drying. Second, PLA was grafted onto the dodecylated lignin via ring opening polymerization. Typically, dodecylated lignin (0.1 g) reacted with lactide (4.9 g) in anhydrous dichloromethane for 1 day (25 °C and N_2_ atmosphere). 1,8-Diazabicyclo [5.4.0] undec-7-ene was used as a catalyst. A series of LP copolymers were synthesized under similar conditions by varying the feeding ratio of lignin and lactide. For instance, LP20 means that the ratio of lignin: lactide is 20: 80.

### Electrospinning of nanofibers

A mixture of PLLA and respective LP copolymer (2: 1 in mass ratio) were dissolved in 1,1,1,3,3,3-hexafluoro-2-propanol to form a solution of 6% (w/v). The solutions were stirred for 24 h to obtain homogeneous solutions. Next, each solution was transferred into 5 ml syringes with a 25-gauge blunt needle. Pumping rate was set at 1 mL/hour and 12 kV of voltage was applied on the needle. The distance between the needle tip to collector was 15 cm. After spinning, the obtained nanofibers were dried in a vacuum oven overnight, and used for materials characterization, cell culture and animal study. Neat PLLA nanofibers were fabricated under the same parameters as control.

### Characterization of electrospun nanofibers

The morphology of the obtained nanofibers was observed by using SEM (6360LA, JEOL, Japan). Samples were sputter-coated with a thin layer of gold before imaging (20 s and 20 mA). Fiber diameter was determined from 50 random measurements per image by using Image J (National Institutes of Health, Bethesda, USA).

The mechanical properties of the PLLA/LP nanofibers were characterized by using uniaxial tensile testing technique (Instron 5943, USA) with a load cell of 10 N load capacity. Fibrous scaffold were cut into a rectangular shape at 5 mm × 30 mm with the support of a paper frame. The thicknesses of the scaffold were about 100 μm. The gauge length was set as 20 mm and tensile speed was 5 mm/min. At least 5 samples were prepared for each composition. Tensile strength and Young’s modulus were calculated based on the stress-strain curve of each sample.

The viscoelastic behavior of the nanofibers was investigated using a dynamic mechanical analysis (DMA, TA Instrument Q800) with tension film clamps. The samples were cut into rectangular shapes with 5 mm width. The gauge length was set at 10 mm and the stress for creep was 0.5 MPa. The test was carried out at 37 °C, and the duration of measurements was determined as 15 min for the creep and 30 min for creep-recovery tests, respectively.

The antioxidant activity of the PLLA/LP fibers was evaluated by using DPPH assay [40, 41]. Fibers (0.2 g) were placed in glass vials. 60 μM DPPH/MeOH solution was prepared, and 20 ml of such solution was added into each vial. DPPH free radical content was measured by monitoring the absorbance changes at 517 nm at each time point. All samples were prepared in triplicate. The antiradical activity was measured as percentage inhibition of free radicals by measuring the decrease in absorbance compared to control solutions.

### BMSCs isolation and culture

All animal experiments were performed based on the Animal Ethics Committee standards of Guangxi Medical University (Protocol Number: SCXK-Gui-2019-0211). BMSCs were extracted from approximately week-old Sprague-Dawley (SD) rats. The BMSCs were cultured in α-minimal essential medium (α-MEM, Biosharp, China) with 10% foetal bovine serum (Tianhang, China) and 1% streptomycin/penicillin (Biosharp, China) in an incubator with 5% CO_2_ at 37 ℃. The medium was replaced every 3 days.

### Determining antioxidant activity

The BMSCs were pre-seeded on PLLA/LP nanofibers with 0.4 mmol/L H_2_O_2_ for 24 h. Cell viability was performed by using a calcein/PI live/dead viability assay kit (Beyotime, China).

### Cell proliferation and adhesion assay

The nanofibrous membranes were cut into 15 mm diameter circles. They were then placed into 24-well plates to sterilize under ultra violet irradiation for 6 h before BMSCs seeding. The BMSCs (2 × 10^4^ cells/well) were seeded onto nanofibrous membranes for cell adhesion and proliferation test. After 7 days, the cell proliferation was evaluated by Cell Counting Kit-8 (CCK-8) assay with a microplate reader (Thermo Fisher Scientific, USA) at 490 nm wavelength. The BMSCs viability was performed by using a calcein/PI live/dead viability/cytotoxicity assay kit (Beyotime, China). The BMSCs morphology on nanofibrous membranes was observed by scanning electron microscope (TESCAN, VEGA3LMU, Czech Republic). Cell cytoskeleton observation was investigated using rhodamine/phalloidin (Invitrogen, USA) and DAPI was used for nuclei staining.

**Table1:** Primers in qRT-PCR analysis

Genes	Forward primers	Reverse primers
SOX9	TCCAGCAAGAACAAGCCACA	CGAAGGGTCTCTTCTCGCTC
col1a1	GATCCTGCCGATGTCGCTAT	GGGACTTCTTGAGGTTGCCA
col2a1	AAGAACAGCATTGCCTAC	TGGTCTTGCCCCACTTAC
ACAN	GAATGGGAGCCAGCCTACAC	GAGAGGCAGAGGGACTTTCG
col10a1	CCAGCCCCAGGACACAATAC	TCCATGTGGTCCTCTCTCCC
GAPDH	TCCAGTATGACTCTACCCACG	CACGACATACTCAGCACCAG

### In vitro* chondrogenic analysis*

To evaluate chondrogenic ability of PLLA/PL nanofibers, The nanofibrous scaffold were cultured in chondrogenic medium with BMSCs, TGF-β1 (PeproTech, USA, 10 ng/mL), 1% insulin-transferrin-selenium solution (Gibco, USA), dexamethasone (Sigma, 100 nM), and ascorbic acid (Sigma, 50 μg/mL) for 21 days. The quantification of GAG was evaluated by DMMB assay and chondroitin sulfate was used as the standard. The DNA content was tested by Hoechst 33,258 method with a fluorescence microplate (Ex/Em = 360/460 nm), and the calf thymus DNA was used as the standard. Eventually, the secretion of GAG was normalized to the total DNA content.

The qRT-PCR was used to evaluate gene expression levels of *Col2a1*, *ACAN,* and *Sox9*. The total RNA content of the BMSCs was isolated by using the HiPure Total RNA Mini Kit (Magen, China), and complementary DNA was synthesized using a PrimeScript™ RT Reagent Kit (Takara, China) accounting to the product manual. The qRT-PCR were conducted with FastStart Universal SYBR Green Master Mix (Roche, Germany) using a real-time PCR system (Roche, LightCycler®480, Germany. The primers in qRT-PCR analysis were shown in Table 1. The gene expression level of chondrogenic differentiation markers was determined by the 2^−ΔΔCt^ method using glyceraldehyde-3-phosphate dehydrogenase (GAPDH) as an internal control.

The secretion of *Col2a1* was evaluated by the immunofluorescence method. After 21 days, the cells were fixed with 1% paraformaldehyde for 30 min and permeabilized with 0.3% Triton X-100 (w/v) for 10 min. After blocked by 5% Bowene Serum Albumin (BSA) for 15 min at 25 °C, the primary antibody of *Col2a1* (1:200 dilution, CST, USA) was added and incubated at 4 °C overnight, and then the secondary antibody *IgG-CY3* (1:100 dilution, BOSTER, China) was incubated for 2 h in the dark. The samples were observed by a fluorescence microscope.

### Animal procedure

18 SD rats (aged 8–10 weeks, 200–300 g) were used to evaluate the effect of the nanofibers in this study. All animal procedures were approved by the Animal Ethics and Welfare Committee of Guangxi Medical University (Protocol Number: SCXK-Gui-2019-0211). The SD rats were anesthetized by intraperitoneal injection with 2% sodium pentobarbital. A cartilage-only defect (2 mm in diameter × 1.5 mm in depth) at the center of the patella groove was made on the chondral surface using a scalpel. Subsequently, the area was covered with the following cells and nanofibers for 6 weeks of treatment with the following: (1) Control group: cartilage defect without treatment. (2) Control group: cartilage defect treated with BMSCs (1 × 10^5^ cells/mm^2^). (3) PLLA/LP30 group: PLLA/LP30 nanofibers (2 mm × 1.5 mm) seeded with BMSCs (1 × 10^5^cells/mm^2^). After surgery, intramuscular cephalexin (50 mg/kg × 3 for 24 h) were administered postoperatively.

### Histological evaluation

The histological study was done by HE staining, safranin O-fast green staining, and immunohistochemical staining (Col2a1). Briefly, the cartilage tissues were fixed with 4% (v/v) paraformaldehyde for 48 h and decalcified with ethylenediaminetetraacetic (EDTA) acid decalcifying solution (Boster, China). It was then embedded in paraffin and cut into serial sections of 3 μm. The sections were dewaxed and detected by using HE, modified safranine O-fast Green cartilage staining kits (Solaibio, China), and immunohistochemical staining kits (ZSGB-BIO, China) based on the product instructions. Images were observed by using optical microscope (Olympus, Japan).

### Statistical analysis

All the data presented are expressed as mean ± standard deviation of the mean. Student’s t-test and one-way ANOVA were used, and differences between the groups are considered statistically significant at p < 0.05.

## Supplementary Information

**Additional file1: Table S1.** Table characterization of lignin-*g*-PLA copolymers. **Table S2.** TGA characterisation table of PLLA and PLLA/PLA-lignin nanofibers. **Figure S1.** ^1^H NMR (CDCl_3_) of the synthesized alkylated lignin. **Figure S2.** ^1^H NMR (CDCl_3_) of the alkylated lignin-*g*-PLA. **Figure S3.** FTIR spectra of lignin and PLA-lignin copolymers. **Figure S4.** Images showing the water contact angles of PLLA and PLLA/PLA-lignin nanofibers. **Figure S5.** Typical stress-strain curves of the PLLA/PLA-lignin nanofibers by tensile test. **Figure S6.** DSC curves of PLA-lignin copolymers. **Figure S7.** DSC curves of PLLA/lignin-PLA nanofibers.

## Data Availability

All the data analyzed throughout this study are included in the article.

## Abbreviations

OA: Osteoarthritis
ECM: Extracellular matrix
TE: Tissue engineering
ROS: Reactive oxygen species
MSCs: mesenchymal stem cells
UV: absorbing ultraviolet
ATRP: atom transfer radical polymerization
PLA: poly (lactic acid)
LP: lignin-PLA
PLLA: Poly-L-lactic acid
DPPH: 1,1-diphenyl-2-picrylhydrazyl
BMSCs: bone mesenchymal stem cells
PI: propidium iodide
SEM: scanning electron microscopy
DAPI: 4',6-diamidino-2-phenylindole
GAG: glycosaminoglycans
DMMB: 1,9-dimethylmethylene blue
qRT-PCR: Quantitative real-time polymerase chain reaction
Col2a1: collagen type II
ACAN: aggrecan
Sox9: SRY (sex determining region Y)-box9
Col1a1: collagen type I
Col10a1: collagen type X
SD: Sprague-Dawley
ICRS: International Cartilage Repair Society
HE: hematoxylin and eosin
DMA: dynamic mechanical analysis
α-MEM: α-minimal essential medium
CCK-8: Cell Counting Kit-8
GAPDH: glyceraldehyde-3-phosphate dehydrogenase
BSA: Bowene serum albumin
EDTA: ethylenediaminetetraacetic

## References

[1] Musumeci G, Castrogiovanni P, Leonardi R, Trovato FM, Szychlinska MA, Di Giunta A, Loreto C, Castorina S (2014). New perspectives for articular cartilage repair treatment through tissue engineering: a contemporary review. World J Orthope.

[2] Balakrishnan B, Joshi N, Jayakrishnan A, Banerjee R (2014). Self-crosslinked oxidized alginate/gelatin hydrogel as injectable, adhesive biomimetic scaffolds for cartilage regeneration. Acta Biomater.

[3] Kotani K, Sakane N, Kamimoto M, Taniguchi N (2011). Levels of reactive oxygen metabolites in patients with knee osteoarthritis. Australas J Ageing.

[4] Mobasheri A, Kalamegam G, Musumeci G, Batt ME (2014). Chondrocyte and mesenchymal stem cell-based therapies for cartilage repair in osteoarthritis and related orthopaedic conditions. Maturitas.

[5] Musumeci G, Castrogiovanni P, Trovato F, Weinberg A, Al-Wasiyah M, Alqahtani M, Mobasheri A (2015). Biomarkers of chondrocyte apoptosis and autophagy in osteoarthritis. Int J Mol Sci.

[6] Tangtrongsup S, Kisiday JD (2018). Modulating the oxidative environment during mesenchymal stem cells chondrogenesis with serum increases collagen accumulation in agarose culture. J Orthop Res®.

[7] Facchini A, Stanic I, Cetrullo S, Borzì RM, Filardo G, Flamigni F (2011). Sulforaphane protects human chondrocytes against cell death induced by various stimuli. J Cell Physiol.

[8] Bhatti FU, Mehmood A, Latief N, Zahra S, Cho H, Khan SN, Riazuddin S (2017). Vitamin E protects rat mesenchymal stem cells against hydrogen peroxide-induced oxidative stress in vitro and improves their therapeutic potential in surgically-induced rat model of osteoarthritis. Osteoarthritis Cartilage.

[9] Bhattacharya I, Saxena R, Gupta V (2012). Efficacy of vitamin E in knee osteoarthritis management of North Indian geriatric population. Ther Adv Musculoskelet Dis.

[10] Guo Y, Tian D, Shen F, Yang G, Long L, He J, Song C, Zhang J, Zhu Y, Huang C, Deng S (2019). Transparent cellulose/technical lignin composite films for advanced packaging. Polymers.

[11] Sugiarto S, Leow Y, Tan CL, Wang G, Kai D (2022). How far is lignin from being a biomedical material?. Bioactive Materials.

[12] Figueiredo P, Lintinen K, Hirvonen JT, Kostiainen MA, Santos HA (2018). Properties and chemical modifications of lignin: towards lignin-based nanomaterials for biomedical applications. Prog Mater Sci.

[13] Zhang Y, Jiang M, Zhang Y, Cao Q, Wang X, Han Y, Sun G, Li Y, Zhou J (2019). Novel lignin–chitosan–PVA composite hydrogel for wound dressing. Mater Sci Eng C.

[14] Ravishankar K, Venkatesan M, Desingh RP, Mahalingam A, Sadhasivam B, Subramaniyam R, Dhamodharan R (2019). Biocompatible hydrogels of chitosan-alkali lignin for potential wound healing applications. Mater Sci Eng C.

[15] Liu R, Dai L, Hu L-Q, Zhou W-Q, Si C-L (2017). Fabrication of high-performance poly(l-lactic acid)/lignin-graft-poly(d-lactic acid) stereocomplex films. Mater Sci Eng C.

[16] Jiang T, Kai D, Liu S, Huang X, Heng S, Zhao J, Chan BQY, Loh XJ, Zhu Y, Mao C, Zheng L (2018). Mechanically cartilage-mimicking poly(PCL-PTHF urethane)/collagen nanofibers induce chondrogenesis by blocking NF–kappa B signaling pathway. Biomaterials.

[17] Gupta C, Washburn NR (2014). Polymer-grafted lignin surfactants prepared via reversible addition-fragmentation chain-transfer polymerization. Langmuir.

[18] Zhang Y, Liao J, Fang X, Bai F, Qiao K, Wang L (2017). Renewable high-performance polyurethane bioplastics derived from lignin–poly (ε-caprolactone). ACS Sustainable Chem Eng.

[19] Kai D, Ren W, Tian L, Chee PL, Liu Y, Ramakrishna S, Loh XJ (2016). Engineering poly (lactide)–lignin nanofibers with antioxidant activity for biomedical application. ACS Sustainable Chem Eng.

[20] Kai D, Jin G, Prabhakaran MP, Ramakrishna S (2013). Electrospun synthetic and natural nanofibers for regenerative medicine and stem cell. Biotechnol J.

[21] Huang Z, Zhang Y, Kotaki M, Ramakrishna S (2003). A review on polymer nanofibers by electrospinning and their applications in nanocomposites. Compos Sci Technol.

[22] Little CJ, Bawolin NK, Chen X (2011). Mechanical properties of natural cartilage and tissue-engineered constructs. Tissue Eng Part B.

[23] Bjørneboe A, Bjørneboe G-EA, Drevon CA (1987). Serum half-life, distribution, hepatic uptake and biliary excretion of α-tocopherol in rats. Biochimica et Biophysica Acta-Lipids and Lipid Metabolism.

[24] Castro RCA, Ferreira IS, Roberto IC, Mussatto SI (2019). Isolation and physicochemical characterization of different lignin streams generated during the second-generation ethanol production process. Int J Biol Macromol.

[25] Sprott MR, Gallego-Ferrer G, Dalby MJ, Salmerón-Sánchez M, Cantini M (2019). Functionalization of PLLA with polymer brushes to trigger the assembly of fibronectin into nanonetworks. Adv Healthc Mater.

[26] Saudi A, Amini S, Amirpour N, Kazemi M, Zargar Kharazi A, Salehi H, Rafienia M (2019). Promoting neural cell proliferation and differentiation by incorporating lignin into electrospun poly(vinyl alcohol) and poly(glycerol sebacate) fibers. Mater Sci Eng C.

[27] Bužarovska A, Blazevska-Gilev J, Pérez-Martnez BT, Balahura LR, Pircalabioru GG, Dinescu S, Costache M (2021). Poly (l-lactic acid)/alkali lignin composites: properties, biocompatibility, cytotoxicity and antimicrobial behavior. J Mater Sci.

[28] Doostmohammadi M, Forootanfar H, Ramakrishna S (2020). Regenerative medicine and drug delivery: progress via electrospun biomaterials. Mater Sci Eng C.

[29] Ugartondo V, Mitjans M, Vinardell MP (2008). Comparative antioxidant and cytotoxic effects of lignins from different sources. Biores Technol.

[30] Xu T, Yao Q, Miszuk JM, Sanyour HJ, Hong Z, Sun H, Fong H (2018). Tailoring weight ratio of PCL/PLA in electrospun three-dimensional nanofibrous scaffolds and the effect on osteogenic differentiation of stem cells. Colloids Surf B.

[31] Liang R, Zhao J, Li B, Cai P, Loh XJ, Xu C, Chen P, Kai D, Zheng L (2020). Implantable and degradable antioxidant poly (ε-caprolactone)-lignin nanofiber membrane for effective osteoarthritis treatment. Biomaterials.

[32] Wang D, Jang J, Kim K, Kim J, Park CB (2019). “Tree to bone”: lignin/polycaprolactone nanofibers for hydroxyapatite biomineralization. Biomacromol.

[33] Ma J, Cai H, Long X, Cheng K, Xu X, Zhang D, Li J (2020). Hyaluronic acid bioinspired polymers for the regulation of cell chondrogenic and osteogenic differentiation. Int J Biol Macromol.

[34] Yaylaci SU, Sen M, Bulut O, Arslan E, Guler MO, Tekinay AB (2016). Chondrogenic differentiation of mesenchymal stem cells on glycosaminoglycan-mimetic peptide nanofibers. ACS Biomater Sci Eng.

[35] Bi W, Deng JM, Zhang Z, Behringer RR, De Crombrugghe B (1999). Sox9 is required for cartilage formation. Nat Genet.

[36] Karabıyık Acar Ö, Bedir S, Kayitmazer AB, Kose GT (2021). Chondro-inductive hyaluronic acid/chitosan coacervate-based scaffolds for cartilage tissue engineering. Int J Biol Macromol.

[37] Heng BC, Cao T, Lee EH (2004). Directing stem cell differentiation into the chondrogenic lineage in vitro. Stem Cells.

[38] Lian C, Wang X, Qiu X, Wu Z, Gao B, Liu L, Liang G, Zhou H, Yang X, Peng Y (2019). Collagen type II suppresses articular chondrocyte hypertrophy and osteoarthritis progression by promoting integrin β1−SMAD1 interaction. Bone Research.

[39] Ren W, Pan X, Wang G, Cheng W, Liu Y (2016). Dodecylated lignin-g-PLA for effective toughening of PLA. Green Chem.

[40] van Lith R, Gregory EK, Yang J, Kibbe MR, Ameer GA (2014). Engineering biodegradable polyester elastomers with antioxidant properties to attenuate oxidative stress in tissues. Biomaterials.

[41] Baheiraei N, Yeganeh H, Ai J, Gharibi R, Azami M, Faghihi F (2014). Synthesis, characterization and antioxidant activity of a novel electroactive and biodegradable polyurethane for cardiac tissue engineering application. Mater Sci Eng C Mater Biolo Appl.

