# Supplementary material for: PLA-lignin nanofibers as antioxidant biomaterials for cartilage regeneration and osteoarthritis treatment
Source: J Nanobiotechnology. 2022 Jul 16;20:327. doi: 10.1186/s12951-022-01534-2 (PMC9287996; doi:10.1186/s12951-022-01534-2)

**PLA-lignin nanofibers as antioxidant biomaterials for cartilage regeneration and osteoarthritis treatment**

Ruiming Liang ^a#^ , Xingchen Yang ^a#^ , Pek Yin Michelle Yew ^b,c#^, Sigit Sugiarto ^b^, Qiang Zhu ^b^, Jinmin Zhao ^a,d^, Xian Jun Loh ^b^, Li Zheng ^a,d*^, Dan Kai ^b,e*^

^a^ Guangxi Engineering Center in Biomedical Materials for Tissue and Organ Regeneration & Collaborative Innovation Center of Regenerative Medicine and Medical Biological Resources Development and Application, Life Sciences Institute, Guangxi Medical University, Nanning, 530021, China

^b^ Institute of Materials Research and Engineering (IMRE), A*STAR, 2 Fusionopolis Way, #08-03 Innovis, 138634, Singapore

^c^ Department of Biomedical Engineering, Faculty of Engineering, National University of Singapore, 117583, Singapore

^d^ Department of Orthopaedics Trauma and Hand Surgery, Guangxi Key Laboratory of Regenerative Medicine, The First Affiliated Hospital of Guangxi Medical University, Guangxi Medical University, Nanning, 530021, China

^e^ Institute of Sustainability for Chemicals, Energy and Environment (ISCE2), A*STAR, 2 Fusionopolis Way, Innovis, #08-03, Singapore 138634

E-mail: zhengli224@163.com; kaid@imre.a-star.edu.sg

Totally 10 pages with 2 tables and 7 figures

Table S1 Table characterization of lignin-*g*-PLA copolymers

| **Sample** | **Feed Ratio** | | | **Mn ^i^** | **Mw ^i^** | **PDI** | **Yield ^ii^** |
| --- | --- | --- | --- | --- | --- | --- | --- |
|  | **Lignin : Lactide** | | | **[ kDa ]** | **[ kDa ]** |  | **[%]** |
| PL5 | 5 | : | 95 | 75.6 | 100.2 | 1.33 | 92 |
| PL10 | 10 | : | 90 | 61.5 | 87.2 | 1.42 | 80 |
| PL20 | 20 | : | 80 | 49.0 | 57.2 | 1.17 | 72 |
| PL30 | 30 | : | 70 | 38.7 | 47.7 | 1.23 | 76 |
| PL40 | 40 | : | 60 | 28.5 | 35.9 | 1.26 | 69 |
| PL50 | 50 | : | 50 | 15.4 | 21.2 | 1.38 | 65 |
| ^i^ Determined by GPC. ^ii^ Isolated yield. | | | | |  |  |  |

Table S2 TGA characterisation table of PLLA and PLLA/PLA-lignin nanofibers

| **Sample** | **T_95_ (°C) ^a^** | **T_max_ (°C) ^b^** | **Residue at 400°C** | **Residue at 500°C** |
| --- | --- | --- | --- | --- |
| PLLA | 311 | 366 | 2.5% | 1.9% |
| PLLA/PL10 | 310 | 360 | 7.1% | 4.5% |
| PLLA/PL20 | 302 | 365 | 8.5% | 6.6% |
| PLLA/PL30 | 292 | 356 | 8.4% | 6.1% |
| PLLA/PL40 | 290 | 354 | 12.9% | 9.8% |
| PLLA/PL50 | 281 | 351 | 15.2% | 11.5% |
| ^a^ Temperature at 5 wt% loss. ^b^ Maximum degradation temperature. | | | |  |

Figure S1 ^1^H NMR (CDCl_3_) of the synthesized alkylated lignin


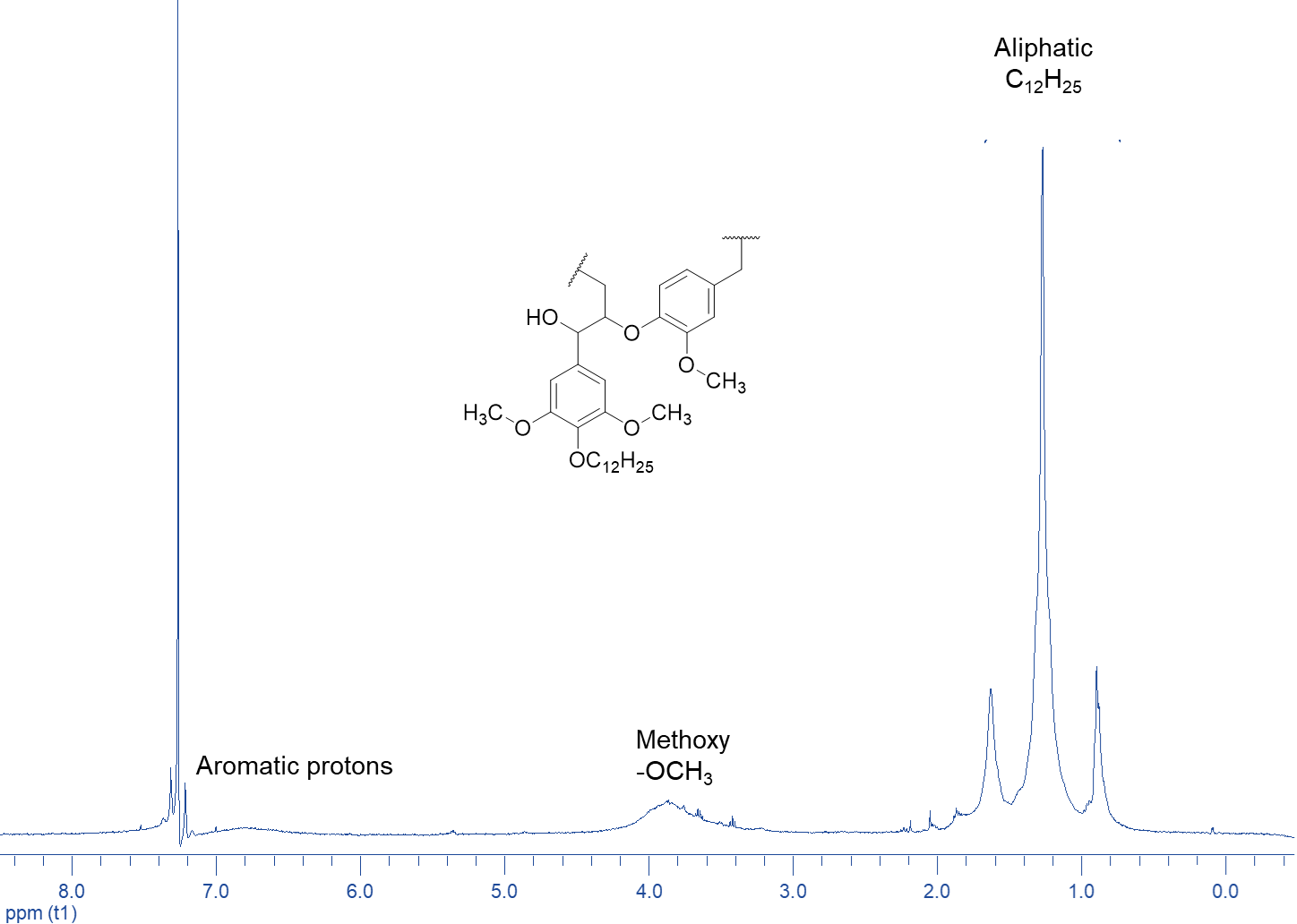


Figure S2 ^1^H NMR (CDCl_3_) of the alkylated lignin-*g*-PLA


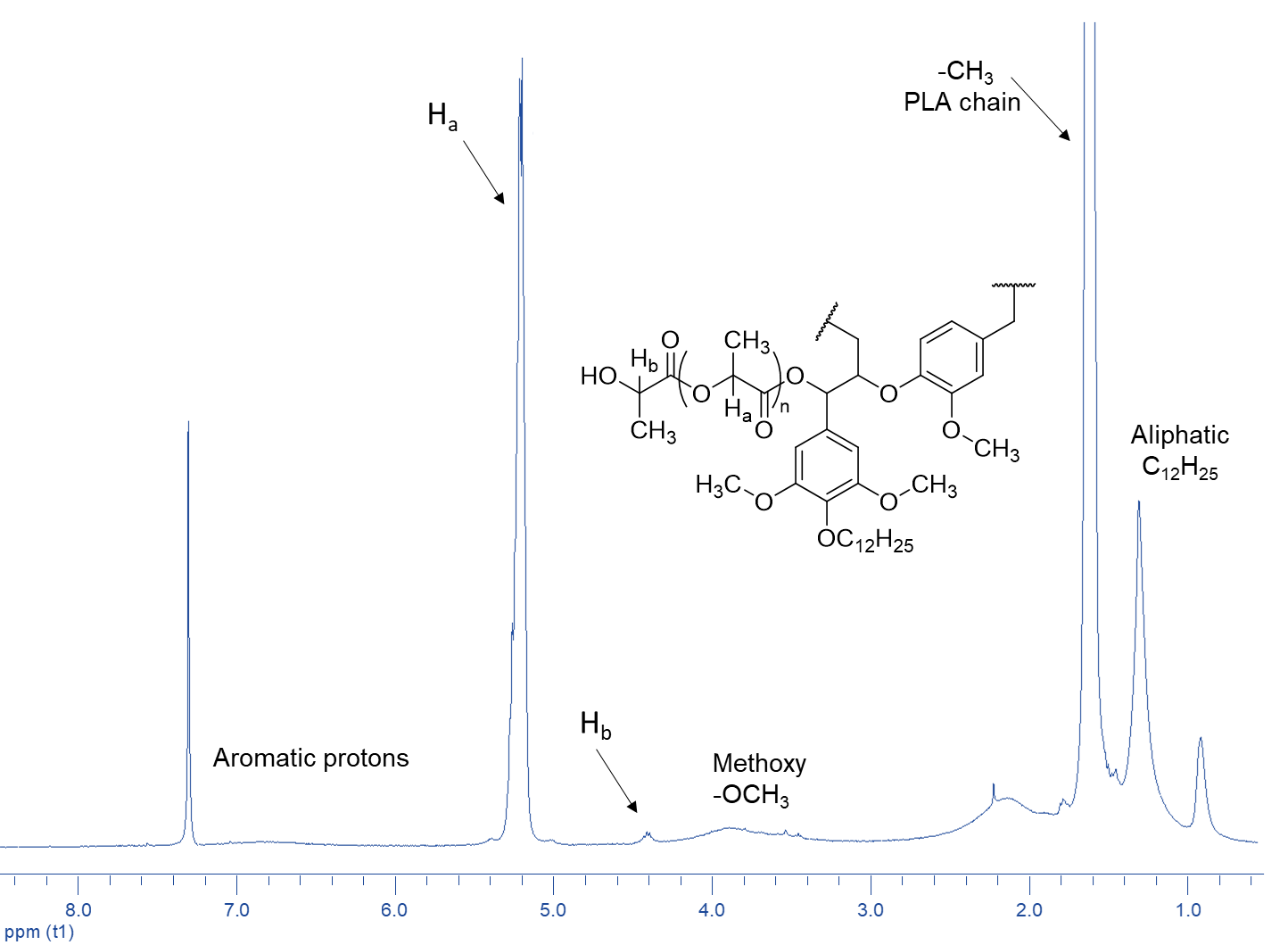


Figure S3 FTIR spectra of lignin and PLA-lignin copolymers.


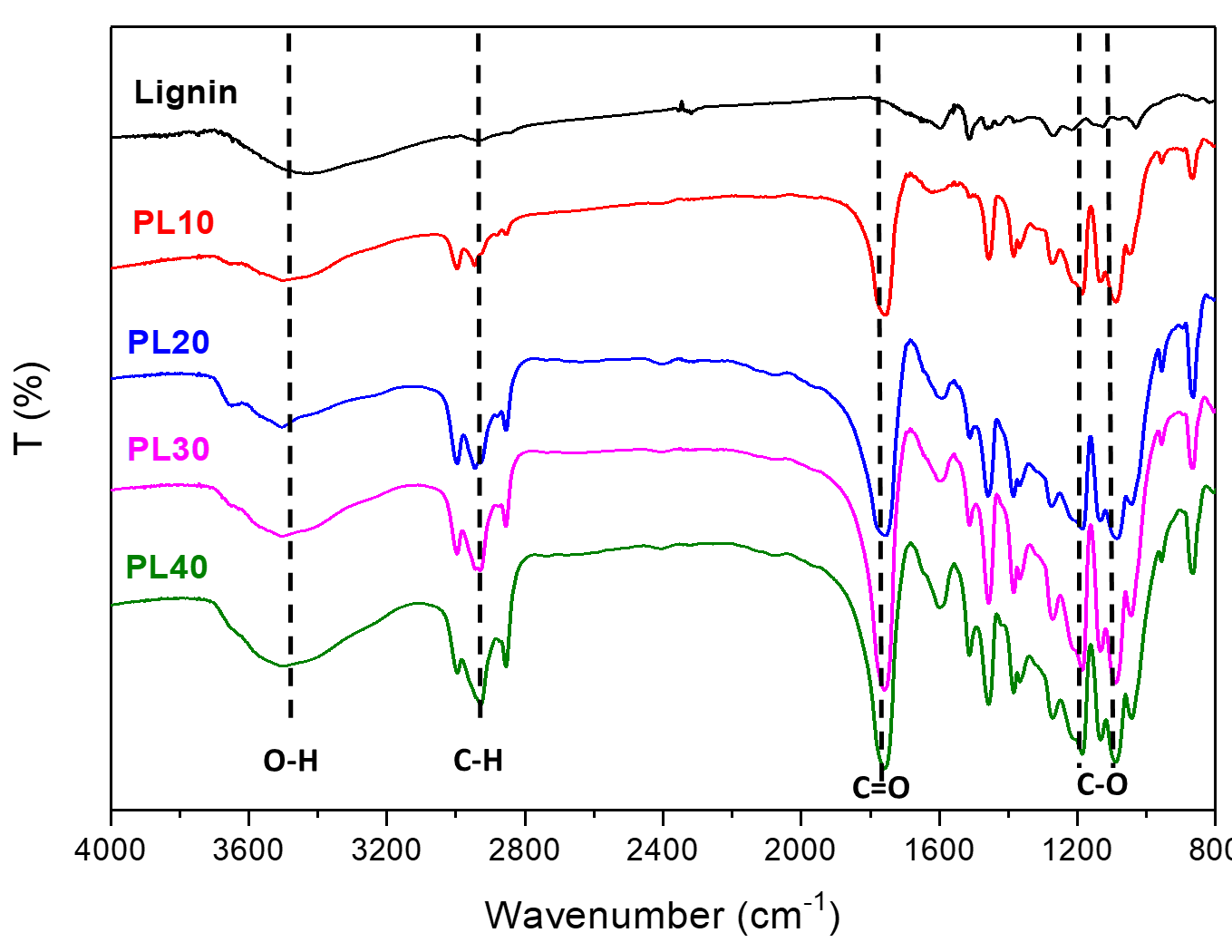


Figure S4 Images showing the water contact angles of PLLA and PLLA/PLA-lignin nanofibers


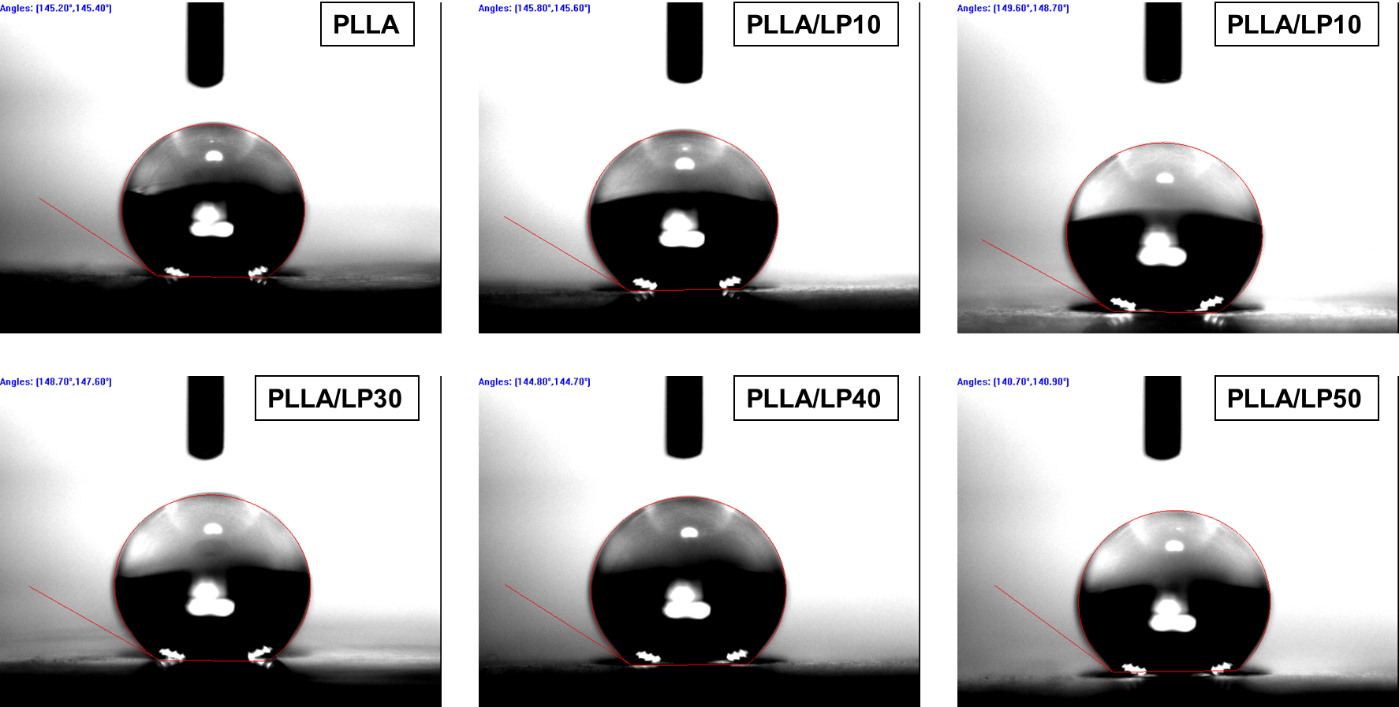


Figure S5 Typical stress-strain curves of the PLLA/PLA-lignin nanofibers by tensile test.

Figure S6 DSC curves of PLA-lignin copolymers

Figure S7 DSC curves of PLLA/lignin-PLA nanofibers


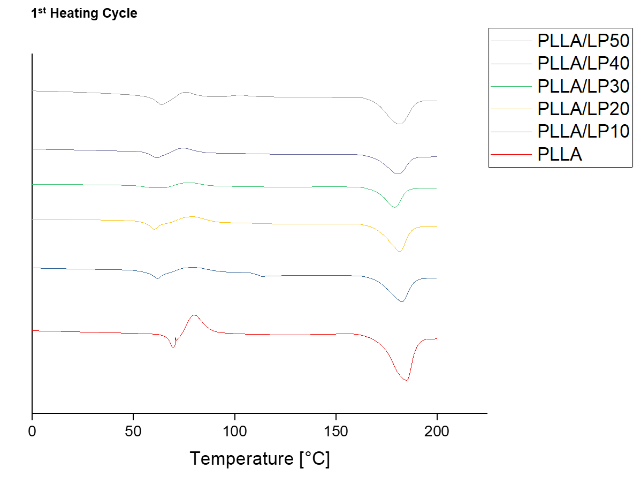

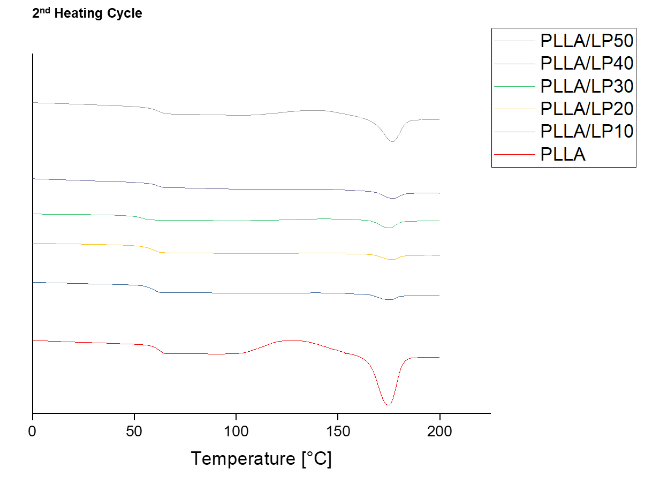

Supplement: Supplementary file 1 — Additional file1: Table S1. Table characterization of lignin-g-PLA copolymers. Table S2. TGA characterisation table of PLLA and PLLA/PLA-lignin nanofibers. Figure S1. 1H NMR (CDCl3) of the synthesized alkylated lignin. Figure S2. 1H NMR (CDCl3) of the alkylated lignin-g-PLA. Figure S3. FTIR spectra of lignin and PLA-lignin copolymers. Figure S4. Images showing the water contact angles of PLLA and PLLA/PLA-lignin nanofibers. Figure S5. Typical stress-strain curves of the PLLA/PLA-lignin nanofibers by tensile test. Figure S6. DSC curves of PLA-lignin copolymers. Figure S7. DSC curves of PLLA/lignin-PLA nanofibers. [file 12951_2022_1534_MOESM1_ESM.docx]
